# Computed tomography texture analysis for the prediction of lateral pelvic lymph node metastasis of rectal cancer

**DOI:** 10.1186/s12957-022-02750-8

**Published:** 2022-09-03

**Authors:** Toshihiro Nakao, Mitsuo Shimada, Kozo Yoshikawa, Takuya Tokunaga, Masaaki Nishi, Hideya Kashihara, Chie Takasu, Yuma Wada, Toshiaki Yoshimoto

**Affiliations:** Department of Digestive and Transplant Surgery, Tokushima University Hospital, 3-18-15 Kuramoto-cho, Tokushima, 770-8503 Japan

**Keywords:** Texture analysis, Computed tomography, Lateral pelvic lymph node, Rectal cancer

## Abstract

**Background:**

This study aimed to investigate the usefulness of computed tomography (CT) texture analysis in the diagnosis of lateral pelvic lymph node (LPLN) metastasis of rectal cancer.

**Methods:**

This was a retrospective cohort study of 45 patients with rectal cancer who underwent surgery with LPLN dissection at Tokushima University Hospital from January 2017 to December 2021. The texture analysis of the LPLNs was performed on preoperative CT images, and 18 parameters were calculated. The correlation between each parameter and pathological LPLN metastasis was evaluated. The texture parameters were compared between pathologically metastasis-positive LPLNs and metastasis-negative LPLNs.

**Results:**

A total of 40 LPLNs were extracted from 25 patients by preoperative CT scans. No LPLNs could be identified in the remaining 19 patients. Eight of the 25 patients had pathologically positive LPLN metastasis. Extracted LPLNs were analyzed by the texture analysis. Pathologically metastasis-positive LPLNs had significantly lower mean Hounsfield unit, gray-level co-occurrence matrix (GLCM) energy, and GLCM Entropy_log2 values, and a significantly larger volume than pathologically metastasis-negative LPLNs. Multivariate analysis revealed that the independent predictive factors for LPLN metastasis were volume (a conventional parameter) (odds ratio 7.81, 95% confidence interval 1.42–43.1, *p* value 0.018) and GLCM Entropy_log2 (a texture parameter) (odds ratio 12.7, 95% confidence interval 1.28–126.0, *p* value 0.030). The combination of both parameters improved the diagnostic specificity while maintaining the sensitivity compared with each parameter alone.

**Conclusion:**

Combining the CT texture analysis with conventional diagnostic imaging may increase the accuracy of the diagnosis of LPLN metastasis of rectal cancer.

## Background

Colorectal cancer is the third most common cancer in men and the second most common cancer in women worldwide, with high morbidity and mortality rates [1]. Furthermore, rectal cancer accounts for 40% of colorectal cancer [2]. Although the outcome of rectal cancer has been improved by total mesorectal excision and neoadjuvant chemoradiotherapy, the local recurrence rates are still as high as 7.4–12.6% [3]. The local recurrence rate of rectal cancer is related to lateral pelvic lymph node (LPLN) metastasis [4], which is reportedly detected in 7.3% of patients with lower rectal cancer [5]. Japanese guidelines consider LPLN metastasis a local condition and recommend LPLN dissection [6], whereas Western guidelines consider LPLN metastasis a systemic disease and recommend neoadjuvant chemoradiotherapy [7]. Regardless of whether the Japanese or Western strategy is performed, it is important to properly assess the condition of the LPLNs prior to treatment.

Currently, the main method used to predict the LPLN metastasis is size-based diagnosis using the largest short-axis diameter of the lymph node on computed tomography (CT) or magnetic resonance imaging (MRI) [8]. However, Ogawa et al. reported that the accuracy of the short-axis diameter on MRI for predicting LPLN metastasis is 77.6% [9], which is not satisfactory.

The texture analysis is an image-processing algorithm that quantifies tissue heterogeneity by evaluating the distribution of the texture coarseness, density, and irregularity within a lesion and is expected to provide a more detailed and quantitative characterization of lesions than visual analysis. Several studies have reported the usefulness of the texture analysis in predicting lymph node metastasis of various cancers [10]. However, to our knowledge, the value of the CT texture analysis in the prediction of LPLN metastasis of rectal cancer has not been reported. The present study aimed to investigate the usefulness of the CT texture analysis in the prediction of LPLN metastasis of rectal cancer.

## Methods

### Patients

This was a retrospective cohort study of 45 patients with rectal cancer who underwent surgery at Tokushima University Hospital from January 2017 to December 2021. The inclusion criteria were lower rectal cancer of clinical stage ≥ T2 or LPLN metastasis in patients with recurrent rectal cancer who underwent LPLN dissection. The exclusion criterion was the absence or unavailability of contrast-enhanced CT images. All patients who underwent preoperative chemotherapy were treated with FOLFOXIRI (irinotecan: 150 mg/m^2^/90 min, leucovorin: 200 mg/m^2^ for 120 min, oxaliplatin: 85 mg/m^2^ for 120 min, 5-fluorouracil: 2400 mg/m^2^ for 44 h) regimen with bevacizumab (5 mg/kg for 90 min) or panitumumab (60 mg/kg for 60 min). All patients who underwent preoperative chemoradiotherapy (CRT) received S-1 orally (80 mg/m^2^/day on days 1–5, 8–12, 15–19, and 22–26) and infusions of oxaliplatin (50 mg/m^2^ on days 1, 8, 15, and 22) and bevacizumab (5 mg/kg on days 1 and 15). The total radiation dose was 40 Gy delivered in daily fractions of 2 Gy via the four-field technique. Surgery was performed 6–9 weeks after completing preoperative CRT.

### CT scan protocol

A 320-detector row CT scanner (Aquilion ONE™/GENESIS Edition, Canon Medical Systems, Tokyo, Japan) was used. The scanning parameters were tube voltage, 120 kV; tube current, auto exposure control; tube rotation speed, 0.5 per second; slice thickness, 0.5 mm; reconstruction kernel, FC14; image reconstruction interval, 0.5 mm; helical pitch, 129; and interpolation method, 180°. Dynamic contrast-enhanced CT was performed using a bolus-tracking technique, where a region of interest (ROI) was placed on the abdominal aorta and the trigger threshold inside the ROI was set at 120 Hounsfield units (HU). The first-phase scan was started 8 s after the threshold was achieved following the administration of contrast material. The second-phase image was obtained 15 s after the first arterial phase. CT was performed within 1 month prior to surgery.

### CT texture analysis

**Fig. 1:**
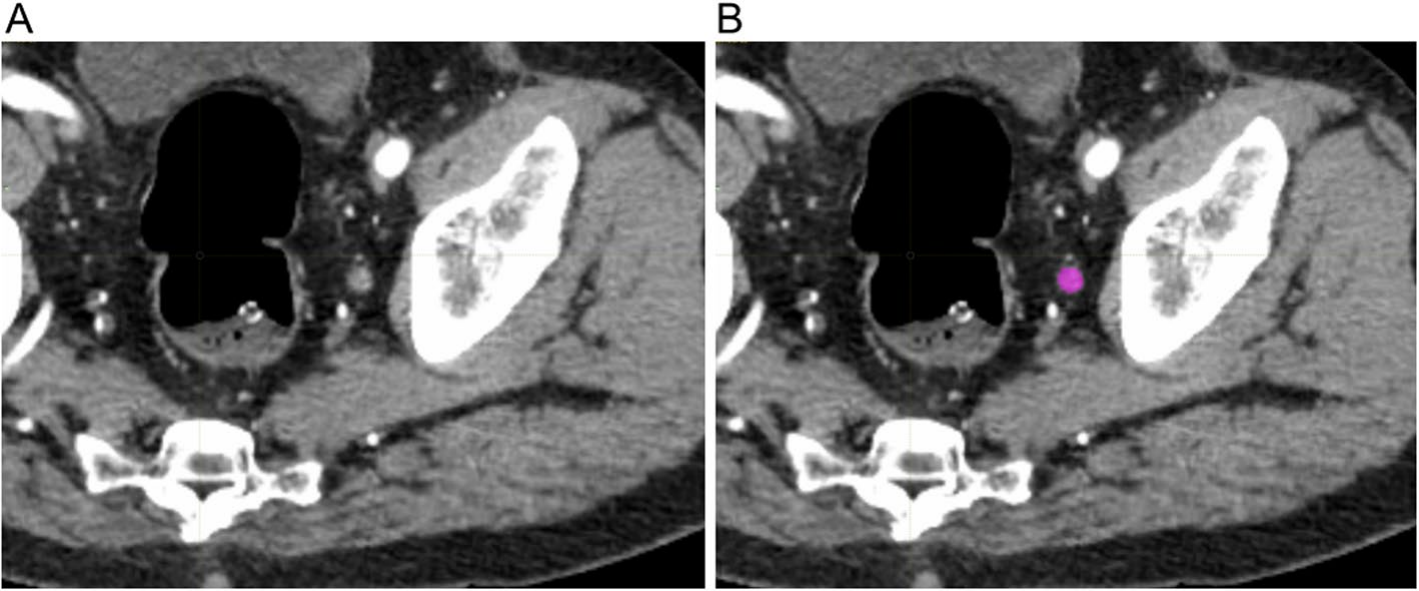
Analysis of the texture parameters on the axial first-phase computed tomography (CT) image that yielded the maximum lateral pelvic lymph node area. **A** An enlarged lateral pelvic lymph node. **B** The region of interest was drawn manually along the outer edge of the lymph node and the texture parameters were extracted

To calculate the texture parameters, DICOM image files of the LPLNs were imported into the software package LIFEx (version 7.1.0, http://www.lifexsoft.org/). All clinical results were blinded during the measurement. The ROI was manually traced on the axial slice of the first-phase CT image that yielded the maximum LPLN area, as already reported (Fig. 1) [11]. Texture parameters of the ROI were automatically extracted using LIFEx software.

Eighteen texture parameters were extracted, including conventional parameters, histogram-based parameters, shape-based parameters, and gray-level co-occurrence matrix (GLCM) parameters. The minimum (HU min), mean attenuation (HU mean), standard deviation (HU std), and maximum values of HU (HU max) were evaluated as conventional parameters. The skewness, kurtosis, entropy_log10, entropy_log2, and energy values were evaluated as histogram-based parameters. The volume and sphericity values were evaluated as shape-based parameters. The homogeneity, energy, contrast, correlation, entropy_log10, entropy_log2, and dissimilarity values were evaluated as GLCM parameters.

### Histopathologic analysis

All patients underwent robot-assisted surgery with LPLN dissection, and all resected tumors and lymph nodes were evaluated by pathologists in accordance with the Seventh Edition of the American Joint Committee on Cancer TNM grading system.

### Statistical analysis

All statistical analyses were performed with EZR (version 1.54) (Saitama Medical Center, Jichi Medical University, Saitama, Japan), a graphical user interface for R (version 4.03) (R Foundation for Statistical Computing, Vienna, Austria) [12]. EZR is a modified version of R commander (version 2.7–1) designed to add statistical functions frequently used in biostatistics. Categorical variables were analyzed with Fisher’s exact test. Continuous variables were analyzed with the Mann–Whitney *U* test. Multivariate analysis was carried out based on the logistic regression model using a forward stepwise selection. The *P* values of < 0.05 were considered statistically significant.

## Results

### Patient characteristics

A total of 45 patients were evaluated. A total of 40 LPLNs were extracted from 25 patients by preoperative CT scans. No LPLNs could be identified in the remaining 19 patients. Eight of the 25 patients had pathologically positive LPLN metastasis. The comparison of patient characteristics between the patients with pathologically positive LPLN metastasis and the patients with pathologically negative LPLN metastasis is summarized in Table 1. The patients with pathologically positive LPLN metastasis showed significantly higher pathological tumor depth of invasion (pT) and pathological lymph node metastasis (pN), and lower differentiation of the tumor than the patients with pathologically negative LPLN metastasis.

**Table 1:** Patient characteristics

	Total (*n* = 25)	LPLNM (*n* = 8)	Non-LPLNM (*n* = 17)	*p*
Age, years	63 (44–84)	65.5 (44–84)	76 (44–81)	0.662
Sex (male/female)	20/5	7/1	13/4	1.000
BMI, kg/m^2^	22.4 (18.6–28.0)	22.85 (19.4–28.0)	22.4 (18.6–26.7)	0.641
ASA-PS (1/2/3)	11/11/3	4/3/1	7/8/2	1.000
Abddominal surgical history	5 (20.0%)	2 (25.0%)	3 (17.6%)	1.000
Preoperative chemotherapy	4 (16.0%)	3 (17.6%)	1 (12.5%)	1.000
Preoperative chemoradiotherapy	5 (20.0%)	2 (11.8%)	3 (37.5%)	0.283
Tumor				
pT (1/2/3/4/LPLNR)	3/5/13/3/1	0/3/2/2/1	3/2/11/1/0	0.049
pN (0/1/2/3/LPLNR)	12/5/0/7/1	0/0/7/1	12/5/0/0	> 0.001
cM (0/1/LPLNMR)	21/3/1	7/0/1	14/3/0	0.188
Differentiation (moderate or high/low)	22/3	5/3	17/0	0.024
L (0/1/X)	7/16/2	1/5/2	6/11/0	0.108
V (0/1/X)	7/15/3	3/3/2	4/12/1	0.263
Surgery				
Procedure (AR/ISR/APR/TPE)	12/3/9/1	6/0/2/0	3/3/10/1	0.051
Robot/Laparoscopy	18/7	6/2	12/5	1.000
taTME	22 (88.0%)	16 (75.0%)	6 (94.1%)	0.231
Diverting stoma	14 (56.0%)			
Operative time, min	479 (240–867)	549.5 (417–678)	413 (240–867)	0.140
Blood loss, ml	65 (0–760)	68 (30–160)	65 (0–760)	0.502
Dissected lateral lymph nodes	12 (2–24)	11 (5–23)	12 (2–24)	0.930

*LPLNM* Lateral lymph node metastasis, *BMI* Body mass index, *ASA-PS* American Society of anesthesiologists physical status, *pT* Pathological tumor depth of invasion, *LPLNR* Lateral pelvic lymph node metastatic recurrence, *pN* Pathological lymph node metastasis, *cM* Clinical distant metastasis, *CR* Complete response, *L* Lymphatic invasion, *V* Venous invasion, *AR* Anterior resection, *ISR* Intersphincteric resection, *APR* Abdominoperineal resection, *TPE* Total pelvic exenteration, *taTME* Transanal total mesorectal excision

### Comparison of texture parameters

Extracted LPLNs were analyzed by texture analysis. Fifteen of the 40 LPLNs showed pathologic metastasis. None of the patients whose LPLNs could not be identified by CT scans had pathologic lymph node metastasis. The texture parameters were compared between pathologically metastasis-positive LPLNs and metastasis-negative LPLNs (Table 2). Pathologically, metastasis-positive LPLNs had significantly lower mean HU, GLCM Energy, and GLCM Entropy_log2 values and a significantly larger volume than pathologically metastasis-negative LPLNs.

**Table 2:** Comparison of the texture parameters of pathologically metastasis-positive and metastasis-negative lateral pelvic lymph nodes

		pLPLN ( +) (*n* = 15)	pLPLN ( −) (*n* = 25)	*p* value
Conventional parameters				
HU min		121.06 ± 18.26	130.73 ± 26.27	0.148
HU mean		21.54 ± 6.17	27.65 ± 6.71	0.012
HU std		173.40 ± 27.76	175.36 ± 26.67	0.386
HU max		106.79 ± 22.33	110.99 ± 29.09	0.665
Histogram-based parameters				
Skewness		3.57 ± 2.77	2.73 ± 0.76	0.201
Kurtosis		0.57 ± 2.77	-0.27 ± 0.76	0.201
Entropy_log10		3.00 ± 0.42	3.18 ± 0.32	0.156
Entropy_log2		0.15 ± 0.05	0.13 ± 0.03	0.173
Energy		1126.56 ± 18.44	1136.12 ± 26.39	0.164
Shape-based parametersHAPE				
Volume(ml)		263.07 ± 334.62	75.60 ± 54.81	0.015
Sphericity		6.60 ± 25.56	7.08 ± 35.40	0.767
GLCM				
Homogeneity		0.04 ± 0.02	0.04 ± 0.01	0.783
Energy		4.70 ± 5.73	6.91 ± 4.60	0.004
Contrast		0.55 ± 0.18	0.50 ± 0.15	0.140
Correlation		1.54 ± 0.20	1.53 ± 0.14	0.472
Entropy_log10		5.11 ± 0.68	5.09 ± 0.46	0.472
Entropy_log2		1.51 ± 0.81	1.95 ± 0.66	0.002
Dissimilarity		0.84 ± 0.07	0.88 ± 0.05	0.119

*pLPLN (* +*)*, pathologically metastasis-positive lateral pelvic lymph nodes; *pLPLN ( −)*, pathologically metastasis-negative lateral pelvic lymph nodes; *HU mini*, minimum of Hounsfield unit; *HU mean*, mean of HU mini, mean Hounsfield unit; *HU std*, standard deviation of Hounsfield unit; *HU max*, maximum of Hounsfield unit; *GLCM*, gray-level co-occurrence matrix

**Fig. 2:**
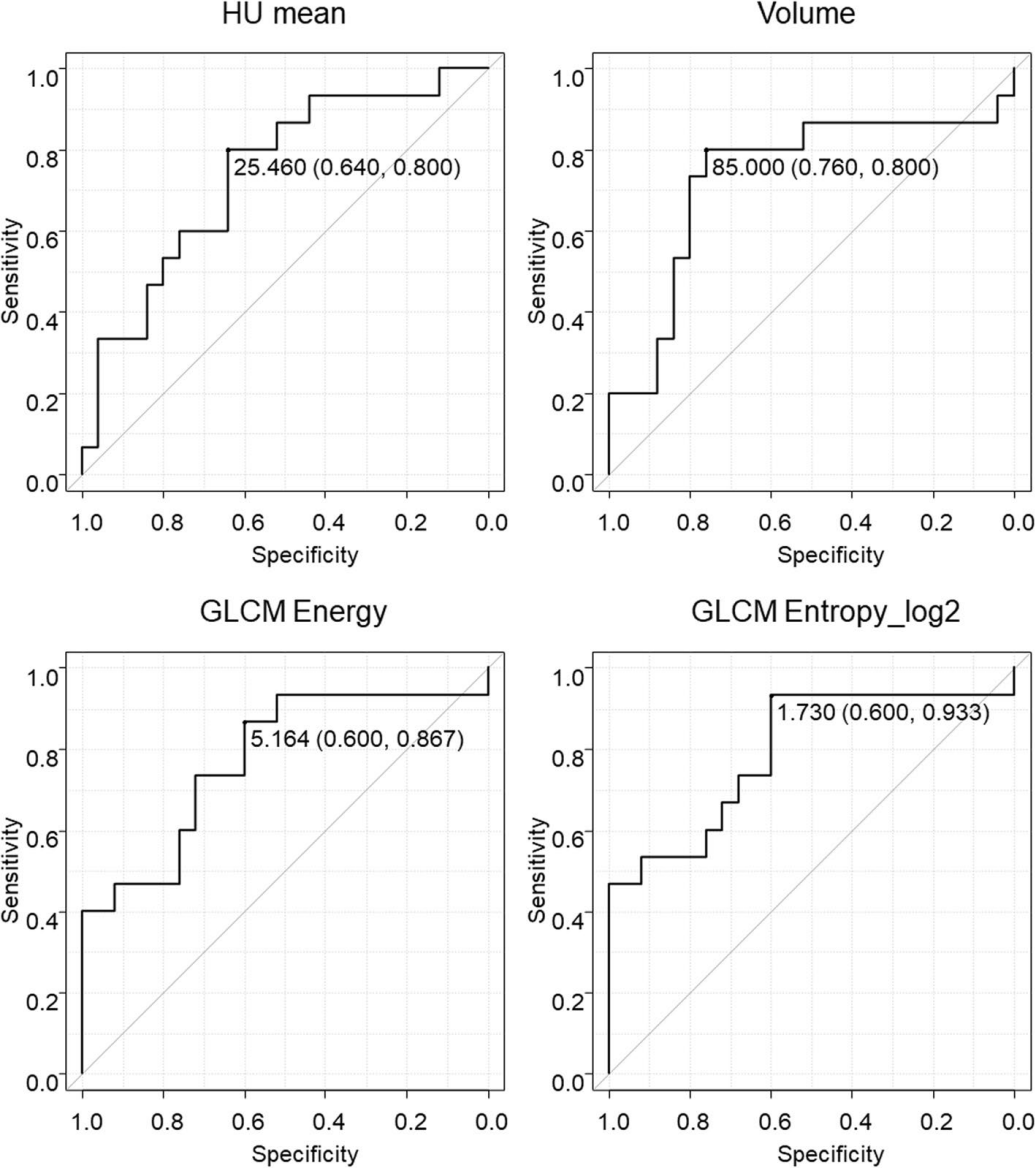
Receiver operating characteristic curves for the prediction of lateral pelvic lymph node metastasis. Curves were created for the parameters with significant differences in univariate analysis

The cutoff values for the prediction of LPLN metastasis were 2.546 for the mean HU, 85.0 for the volume, 5.164 for the GLCM Energy, and 1.73 for the GLCM Entropy_log2; the areas under the receiver operating characteristic curves for each parameter were 0.739, 0.773, 0.773, and 0.792, respectively (Fig. 2).

### Multivariate analysis

The parameters that showed significant differences in univariate analysis were divided into two groups by cutoff values, and multivariate analysis was performed. Volume and GLCM Entropy_log2 were identified as independent predictive factors for LPLN metastasis (Table 3). The sensitivity and specificity of a positive result for both factors were 66.7% and 84.0%, respectively. The sensitivity and negative predictive value of a negative result for both factors were 100%, respectively.

**Table 3:** Multivariate analysis of the texture parameters

	Volume	GLCM Entropy_log2
Cutoff value	> 5.164	< 1.73
AUC	0.773	0.792
Sensitivity (%)	80.0	93.3
Specificity (%)	76.0	60.0
Odds ratio	7.81	12.7
95% CI	1.42–43.1	1.28–126.0
*p* value	0.018	0.030

*GLCM* Gray-level co-occurrence matrix, *AUC* Area under the receiver operating characteristic curve, *CI* Confidence interval

## Discussion

The present study identified volume (a conventional parameter) and GLCM Entropy_log2 (a texture parameter) as independent predictive factors for LPLN metastasis. The cutoff value of the volume was 85.0 ml, with a sensitivity and specificity of 80.0% and 76.0%, respectively. Some studies have reported the size-based diagnosis of LPLN metastasis on CT or MRI. Sato et al. reported that the maximum short-axis LPLN diameter was significantly larger in metastasis-positive cases than in metastasis-negative cases [13]; the cutoff value was 7.0 mm, with a sensitivity and specificity of 100.0% and 84.2%, respectively. Ishibe et al. reported that the sensitivity and specificity of the diagnosis of LPLN metastasis using a cutoff value of the LPLN diameter of 10 mm on MRI were 43.8% and 98.5%, respectively [14]. However, in actual clinical practice, size-based diagnosis of LPLN metastasis has limitations because even lymph nodes smaller than the cutoff value may be positive for metastasis on histopathological examination.

The present study showed that the GLCM Entropy_log2 value may be useful in the diagnosis of LPLN metastasis using CT texture analysis. The cutoff value of the GLCM Entropy_log2 was 1.730, and the sensitivity and specificity were 93.3% and 60.0%, respectively. Texture analysis has recently been widely applied in diagnostic imaging. Liu et al. evaluated a total of 1054 lymph nodes in patients with colorectal mucinous adenocarcinoma and demonstrated that some CT texture parameters were of significance in differentiating lymph node metastasis [15]. Song and Yin evaluated 108 patients with rectal cancer and demonstrated that texture features obtained from T2-weighted MRI may be valuable for identifying lymph node invasion [16]. He et al. evaluated 199 patients with colorectal cancer and developed machine-learning models for the prediction of regional lymph node metastasis based on 18F-fluorodeoxyglucose positron emission tomography (PET)/CT and PET-based radiomic features [17]. However, to the best of our knowledge, there have been no reports of texture analysis for predicting LPLN metastasis of rectal cancer.

The combination of the volume and the GLCM Entropy_log2 value showed better predictive ability for LPLNM in this study. Following the results of this study, we consider that positive for both factors is an absolute indication o LPLND, that positive for either one of the indicators is a relative indication of LPLND, and that a negative for both factors indicates the possibility of omitting LPLND.

The present study had some limitations. First, the sample size was small. Smaller sample sizes may affect the reliability of results due to increased variability and possible bias. Second, this study included patients who underwent preoperative chemotherapy or chemoradiotherapy. Zhang et al. have reported that histogram and GLCM features were associated with the response to chemotherapy for liver metastasis of colorectal cancer [18]. Liu et al. also reported that GLCM features were associated with the response to chemoradiotherapy for locally advanced rectal cancer [19]. Preoperative treatment may have affected the parameters of the texture analysis in this study as well. Third, the study was a retrospective study. A prospective study is needed to determine the accuracy of the results.

## Conclusion

In the present study, the combination of the volume and the GLCM Entropy_log2 value showed better predictive ability for LPLNM in this study. The specificity was improved by the positivity of both factors, and the sensitivity was improved by the negativity of both factors. Combining texture analysis with conventional diagnostic imaging may lead to a more accurate prediction of LPLN metastasis of rectal cancer.

## Data Availability

The datasets generated during and/or analyzed during the current study are available from the corresponding author on reasonable request.

## Abbreviations

LPLN: Lateral pelvic lymph node
CT: Computed tomography
MRI: Magnetic resonance imaging
ROI: Region of interest
HU: Hounsfield units
GLCM: Gray-level co-occurrence matrix
PET: Positron emission tomography
